# Modeling and experimental evaluation of biochar-mediated biofiltration for hydrogen sulfide capture from biogas

**DOI:** 10.1371/journal.pone.0339352

**Published:** 2025-12-31

**Authors:** Mohsen Zarei, Mohammad Reza Bayati, Abbas Rohani, Mohammadali Ebrahimi-Nik, Bijan Hejazi

**Affiliations:** 1 Department of Biosystems Engineering, Faculty of Agriculture, Ferdowsi University of Mashhad, Mashhad, Iran; 2 Department of Chemical Engineering, Faculty of Engineering, Ferdowsi University of Mashhad, Mashhad, Iran; Universidad Nacional de Chimborazo, ECUADOR

## Abstract

Efficient hydrogen sulfide (H₂S) removal is critical for enhancing biogas quality and enabling its utilization. This study proposes a novel, advanced modeling method for predicting H₂S removal efficiency (RE) in biofilters. This method requires fewer assumptions and less input data compared to traditional approaches. To investigate the influence of key parameters on RE, laboratory experiments were conducted using a biochar packed-bed biofilter. The experiments varied moisture content (MC), empty-bed residence time (EBRT), and influent H₂S concentration. All three variables significantly impacted RE, with optimal removal achieved using biochar with 30% MC, 60 seconds EBRT, and 180 ppmv H₂S. Multiple linear regression (MLR) and support vector machine (SVM) techniques were employed to model RE, achieving high accuracy with R² values of 0.97 and 0.99, respectively. These models effectively predicted H₂S removal in the biofilter, demonstrating the reliability and effectiveness of the proposed advanced modeling approach. Additionally, genetic algorithms were used for optimization, suggesting the feasibility of attaining 90–95% RE across a range of H₂S concentrations. Overall, this study introduces a groundbreaking modeling method for H₂S RE in biofilters, offering a practical solution for efficient biogas desulfurization.

## 1. Introduction

Hydrogen sulfide (H₂S), a toxic and highly corrosive gas produced from the degradation of sulfur compounds, is commonly present in biogas at concentrations of up to 10,000 ppmv, even though biogas provides significant environmental benefits [1]. The presence of H₂S lowers biogas quality, creates health and environmental risks, and corrodes equipment, making its effective removal essential for safe utilization and sustainable energy production [2]. However, conventional upgrading technologies for H₂S removal remain costly, particularly for small- and medium-scale applications [3].

H₂S is a common contaminant in biogas that corrodes equipment and reduces the efficiency of energy conversion systems, creating significant operational challenges [4]. The acceptable H₂S concentration depends on the technology and its application, and for combustion processes it should be maintained at 200–300 ppmv [5,6]. During combustion, H₂S oxidizes to sulfur dioxide (SO₂), which accelerates metal corrosion at high temperatures and poisons catalysts used in steam reforming [7]. Several techniques have been developed for H₂S removal from biogas, and their selection is influenced by factors such as gas composition, required removal efficiency, end use, and operating conditions [8]. Conventional methods—including wet oxidation [9], scrubbing [10], adsorption [11], and biological oxidation [12]— are well established. Among these, adsorption is particularly attractive because it is cost-effective, easy to operate, and provides high removal efficiency [13]. Activated carbon is one of the most effective adsorbents, but its high cost limits large-scale use [14]. Biochar offers a low-cost alternative with a high surface area, abundant pores, and functional groups that enhance H₂S capture [3]. Biochars derived from waste materials, such as biosolids, also contain mineral components that further improve their adsorption performance [15]. These properties make char a promising and sustainable adsorbent for H₂S removal as well as other environmental applications, including nutrient recovery and heavy metal removal [16]. The removal of H₂S by biochar occurs in two sequential steps. First, H₂S is physically adsorbed onto the biochar surface. Second, in the presence of water and metal impurities, the adsorbed H₂S is oxidized to sulfur compounds [17]. Biochar is a cost-effective and sustainable adsorbent for removing H₂S from biogas. Its high surface area, porous structure, alkaline properties, and mineral content enhance both physisorption and chemisorption, making it a strong alternative to conventional adsorbents [3]. The efficiency of biofilters is largely determined by microbial activity, which is influenced by operational parameters such as bed moisture, empty bed residence time, nutrient availability, and pH [18]. Filter bed structure and composition are also critical, as factors like particle size, porosity, and nutrient content directly affect microbial attachment, growth, and overall degradation performance [19]. Incorporating biochar into the packing material has been shown to enhance microbial activity, improve mass transfer, and stabilize biofilm formation, resulting in substantially higher H₂S removal compared to conventional packing media [20]. The efficiency of biofiltration systems is strongly influenced by operational factors, including inlet H₂S concentration, empty bed residence time, moisture content, pH, and the physicochemical properties of the packing material. Optimizing these parameters is essential to achieve stable and efficient H₂S removal [21,22]. Consequently, designing and optimizing biochar-based filters is essential, underscoring the significance of this study.

Accurate prediction of biofilter performance is crucial for optimizing H₂S removal and ensuring stable operation [23]. Traditional mathematical models, including dynamic and steady-state approaches, support process design and optimization but often require extensive data and assumptions, limiting their practical applicability [24]. Simple models that capture key system features can provide reliable predictions with minimal overfitting, while more complex models may be needed for higher accuracy [25]. Advanced computational techniques, such as fuzzy logic and artificial neural networks (ANNs), have demonstrated the ability to model non-linear relationships in biofilter systems [26]. Machine learning methods, particularly support vector machines (SVM), can handle high-dimensional datasets and identify complex patterns, providing precise predictions for H₂S removal under varying operational conditions [27]. Multiple linear regression (MLR) remains a widely used approach for practical biofilter modeling and prediction [28]. Despite these developments, no standardized biofiltration model exists, highlighting the need for simplified yet effective predictive tools for real-world applications [24].

In this study, we address this gap by applying both MLR and SVM to predict H₂S removal efficiency in biochar-packed biofilters. Laboratory experiments investigated three key variables: empty bed residence time (EBRT), biochar bed moisture content (MC), and influent H₂S concentration. These experiments allowed the development and validation of predictive models, followed by optimization to determine conditions that maximize H₂S removal. As shown in Table 1, our results demonstrate the effectiveness of biochar as a packing material and the superior predictive capability of MLR and SVM compared to other biofiltration systems reported in the literature. Overall, this work introduces a data-driven framework for H₂S biofiltration, advancing sustainable and environmentally friendly strategies for industrial emission control.

**Table 1:** Comparative evaluation of biofilter bed materials and modeling approaches for gas-phase pollutant removal in biofiltration systems.

Bed type	Gas removed	Model	Max.RE	R^2^	Ref.
Mixture of cornstalk and press mud	Methyl ethyl ketone	Ottengraf–van den Oever	97%	_	[37]
Mixture of compost and wood chips	Benzene and xylene	ANN	70%	0.94	[18]
Compost	Trichloroethylene	ANN	89%	0.99	[38]
Fungi-cultured bio trickling filter in the presence of rhamnolipid	Styrene vapors	ANN	99%	0.99	[39]
Inorganic diatomaceous earth packing pellets	Toluene-styrene mixture	ANN	90%	0.99	[40]
Biochar	H_2_S	MLR & SVM	95%	0.97& 0.99	Current study

## 2. Materials and methods

### 2.1. Production of biogas through anaerobic digestion

**Fig 1:**
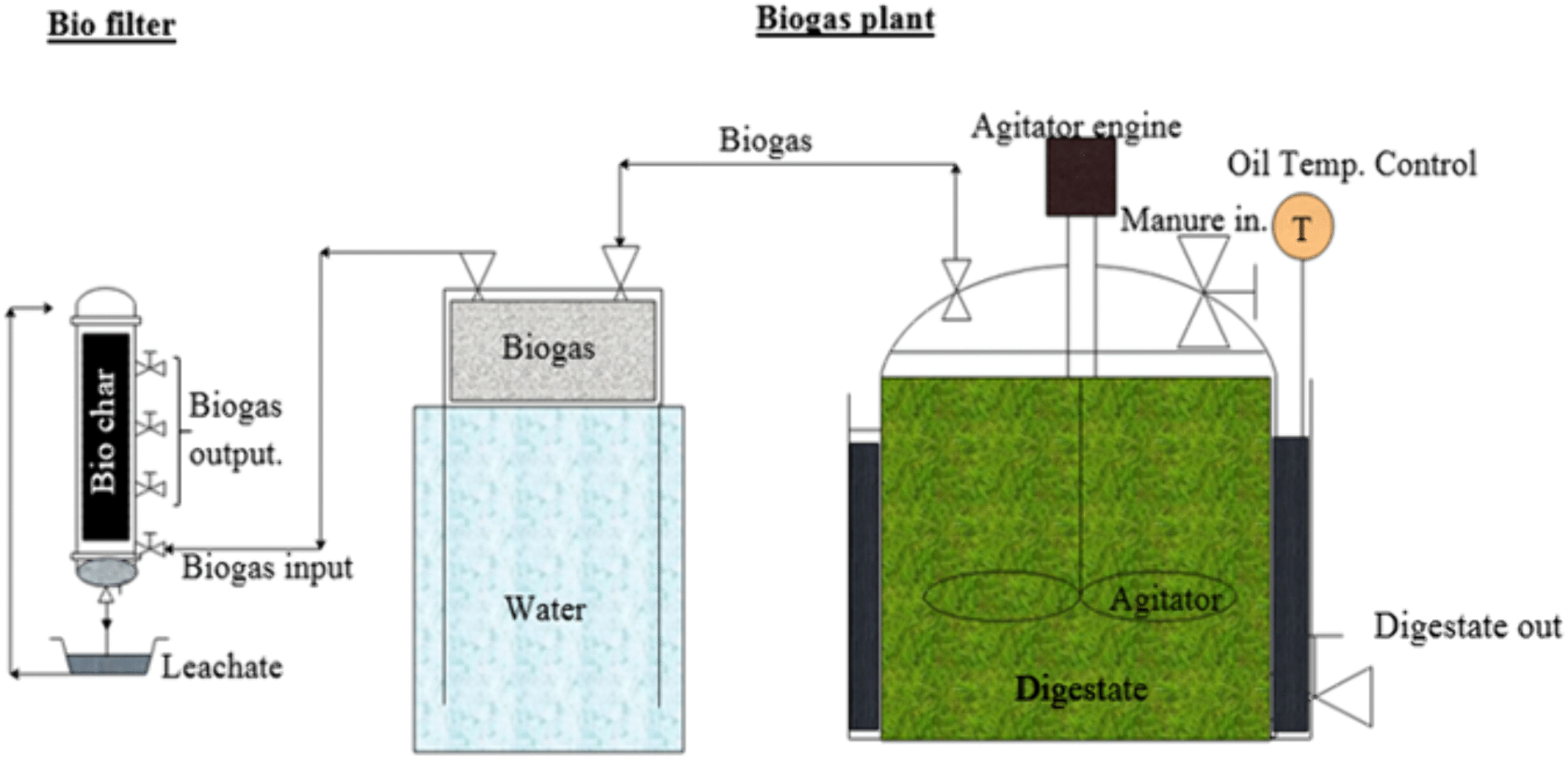
Schematic diagram of the anaerobic digester setup, gas storage tank, and biofilter system for biogas production.

Biogas was produced using a 200-liter anaerobic digester with a diameter of 46 cm and a height of 80 cm (Fig 1). Cow manure was used as the feedstock, and the digester achieved a maximum daily gas production of 72 liters. The produced gas was collected in a storage tank and then gradually introduced into the biofilter. The composition of the biogas was analyzed with a biogas analyzer (Multitec 545, Sewerin, Germany). The analyzer measured methane (CH₄), carbon dioxide (CO₂), and hydrogen sulfide (H₂S). The average composition of the biogas was 60% CH₄, 40% CO₂, and 90–180 ppmv H₂S. The biogas analyzer measured only methane (CH₄), carbon dioxide (CO₂), and hydrogen sulfide (H₂S). Although biogas contains minor components such as hydrogen, ammonia, oxygen, and nitrogen [29], these were not included in the present study because the focus was specifically on H₂S removal in the biofilter.

### 2.2. Biofilter system design and operation

The biofilter system, illustrated in Fig 1, received biogas from a storage tank. It consisted of a PVC column (6 cm diameter, 70 cm height) packed with grape wood biochar. Bed moisture was maintained by spraying tap water onto the top surface, while the drained water was collected in a reservoir and recirculated back to the bed. The biofilter was operated at ambient temperature, ranging from 18 to 23 °C, during all experiments.

### 2.3. Biochar selection and preparation for biofilter operation

Cylindrical biochar particles produced from grape wood were used as the packing material in the biofilter. The biochar was obtained by pyrolysis at 550 °C for two hours under atmospheric pressure. The particles had a height of 5–15 mm and a diameter of 2–5 mm.

The physical and chemical properties of biochar were not measured in this study due to resource limitations; instead, the analysis was based on the most closely related published research. The proximate and elemental composition of comparable grape wood biochar, reported by Nunes et al. (2021), was as follows: volatiles 33.90 ± 0.59%, ash 7.28 ± 0.06%, and fixed carbon 58.82 ± 0.61%. The elemental composition included C (73.53 ± 0.11%), H (4.64 ± 0.05%), N (1.20 ± 0.02%), and O (20.62 ± 0.14%) [30]. Prelac et al. (2023) further reported that biochar produced under similar conditions had a pH of 8.67 ± 0.04 and an electrical conductivity of 2.63 ± 0.29 mS cm ⁻ ¹ [31]. These properties suggest that the material is suitable for biofiltration, as they support stability, nutrient retention, and microbial activity.

This study examined the effects of three key operational factors on the biofiltration process: bed moisture content, empty bed residence time (EBRT), and the initial hydrogen sulfide (H₂S) concentration

#### 2.3.1. *Bed moisture content (MC).*

The moisture content of the biochar bed was determined by weighing a sample and oven-drying it at 104 °C for 24 h. MC was calculated on a wet basis using Equation 1 [32].


$$MC=\frac{W_{w}-W_{d}}{W_{w}}\times \text{1}\text{0}\text{0}$$ (1)

where W_w_ and W_d_ are the wet and dry weights of the biochar (g), respectively. To evaluate the effect of MC on biofilter performance, the bed was prepared at three distinct moisture levels: highly moist (90%), medium moist (70%), and dry (30%) [33,34]. At each level, moisture content was regularly monitored and adjusted as required during the experiments. H₂S removal was measured separately for each condition, capturing the influence of MC over time as the bed naturally dried. These levels correspond to fully wet, intermediate, and dry bed conditions.

#### 2.3.2. Empty bed residence time (EBRT).

The empty bed residence time (EBRT) was calculated using Equation 2 [35]:


$$EBRT=\frac{V_{f}}{Q}$$ (2)

where V_f_ is the total volume of the biofilter column (m³) and *Q* is the inlet biogas flow rate (m³/s). Different EBRT levels were achieved by adjusting the column height or by collecting samples at various heights along the column (see Fig 1)

#### 2.3.3. *Changes in H*_*2*_*S concentration in the biogas.*

The biofilter’s performance was evaluated by varying the inlet H₂S concentration. This was achieved by adjusting the feeding time of the anaerobic digester with fresh cow manure. At the beginning of each batch, H₂S levels were highest and gradually decreased as digestion progressed. Operating the digester in batch mode allowed natural variations in H₂S generation, providing different inlet concentrations for assessing removal efficiency (RE). RE was calculated using Equation (3) [36] and considered the dependent variable in the modeling.


$$RE\;[\%]=\frac{C_{i}-C_{o}}{C_{i}}$$ (3)

where, C_i_ and C_o_ are the H₂S concentrations in the inlet and outlet gas streams of the biofilter, respectively (ppmv).

### 2.4. Modeling H_2_S removal efficiency in biofilters using multiple linear regression

Modeling is essential for predicting and interpreting the performance of biofiltration systems. It enables the identification of interactions among operating parameters and supports the optimization of removal efficiency under varying conditions. Table 1 summarizes previous studies that used different packing materials and modeling approaches, including ANN and mechanistic models, for gas-phase pollutant removal. These studies demonstrate that predictive models are effective tools for explaining biofilter performance and guiding practical applications. Building on this evidence, the present work employs multiple linear regression (MLR) to model and predict H₂S removal efficiency in a biochar-packed biofilter. This approach provides a quantitative framework to assess the influence of key operational factors.

Multiple linear regression (MLR) was applied to model and predict H₂S removal efficiency in a biofilter packed with biochar. The independent variables were bed moisture content (MC), empty bed residence time (EBRT), and the inlet H₂S concentration. The MLR model was expressed as follows (Eq. 4):


$$y=\beta_{\text{0}}+\beta_{\text{1}}x_{\text{1}}+\beta_{\text{2}}x_{\text{2}}+\beta_{\text{3}}x_{\text{3}}+\beta_{\text{1}\text{2}}x_{\text{1}}x_{\text{2}}+\beta_{\text{1}\text{3}}x_{\text{1}}x_{\text{3}}+\beta_{\text{2}\text{3}}x_{\text{2}}x_{\text{3}}+\beta_{\text{1}\text{1}}x_{\text{1}}^{\text{2}}+\beta_{\text{2}\text{2}}x_{\text{2}}^{\text{2}}+\beta_{\text{3}\text{3}}x_{\text{3}}^{\text{2}}+\varepsilon$$ (4)

Here, *x*_*i*_ represents the linear independent variables, *x*_*ij*_ denotes the two-factor interaction (2FI) variables obtained by multiplying two different independent variables, and *x*_*ii*_ indicates the quadratic terms. The coefficients are defined as follows: β₀ is the intercept, β_i_ are the linear coefficients, β_ij_ are the 2FI (interaction) coefficients, and β_ii_ are the quadratic coefficients. The random error vector (ε) was calculated using Eq. 5:


$$\varepsilon =Y-\beta X=Y-\hat{Y}$$ (5)

Coefficient vectors were estimated using the least-squares method, which minimizes the sum of squared errors. The statistical significance of the coefficients was assessed using t-tests, and the overall significance of the model was evaluated with F-tests. An analysis of variance (ANOVA) table was constructed to examine the effect of each factor. Factors with p-values greater than 0.05 were excluded, and the final model was established based on the significant variables.

### 2.5. Modeling H_2_S removal efficiency in biofilters using support vector machine

Support Vector Machine (SVM) is a supervised learning algorithm originally developed for classification. However, it has been successfully adapted for regression problems through Support Vector Regression (SVR) [41]. In regression, SVM does not separate classes; instead, it identifies an optimal hyperplane that best fits the relationship between input and output variables within a defined error margin.

**Fig 2:**
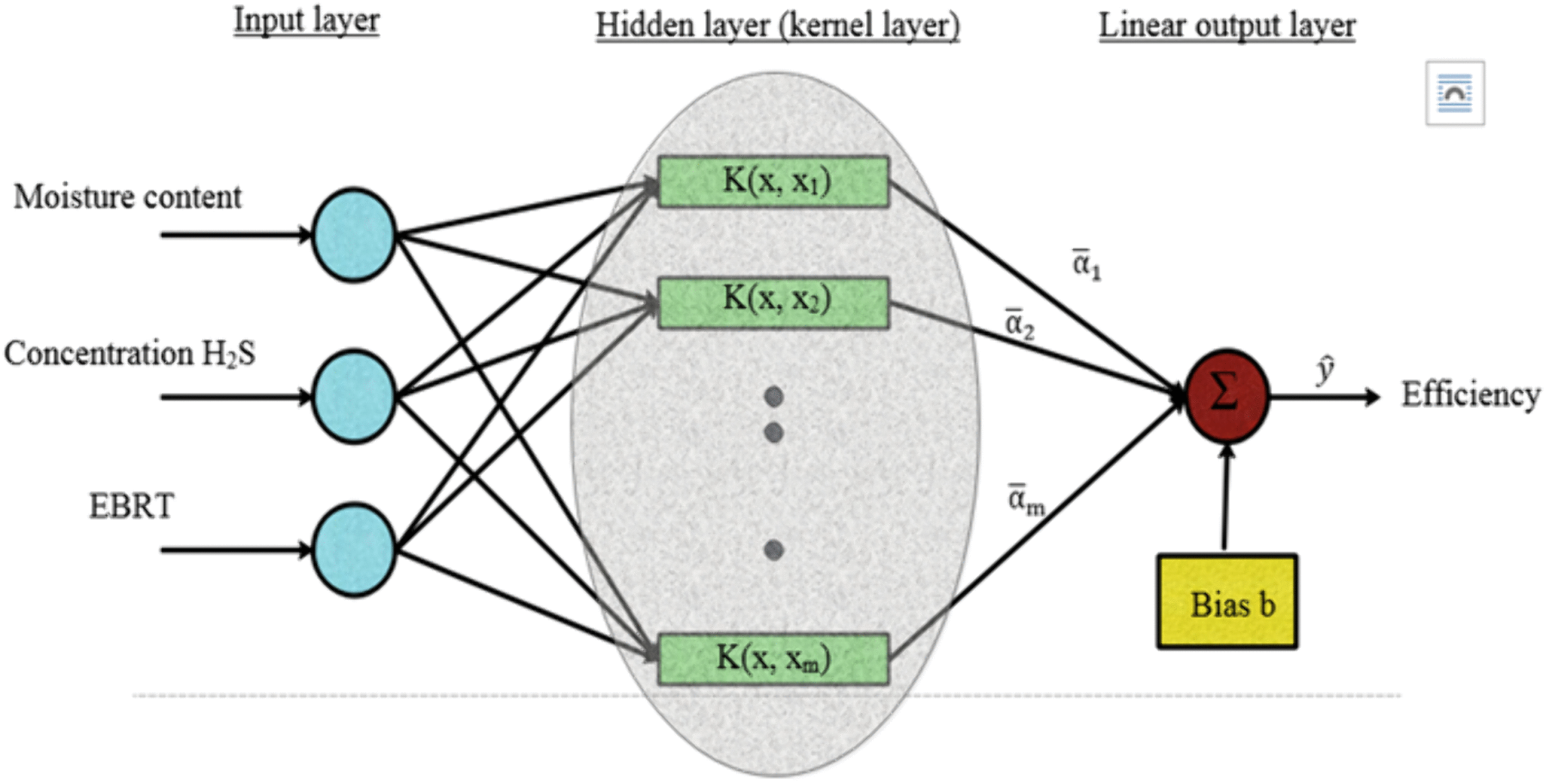
Support vector machine (SVM) model architecture for predicting H2S removal efficiencies.


In this study, the least squares support vector machine (LS-SVM), a computationally efficient variant of SVR, was used to predict H₂S removal efficiency (RE), which is a continuous quantitative variable. The independent variables included bed moisture content (MC), empty bed residence time (EBRT), and inlet H₂S concentration. LS-SVM applies kernel functions to capture complex, non-linear relationships between the inputs and the target output. This approach provides flexibility and accurate prediction of continuous process variables, making it suitable for biofilter studies [42,43]. The structure of the SVM model used in this study is illustrated in Fig 2. The LS-SVM optimization problem can be expressed as:

$$\;Minimize\;J(W,b,e)=\frac{\text{1}}{\text{2}}{\left\|W\right\|}^{\text{2}}+\frac{\text{1}}{\text{2}}{\left\|e\right\|}^{\text{2}}$$

Subject to Y=WX+b+e (6)

where *W* is the weight vector, *b* is the bias term, *X* is the feature matrix, *Y* is the target vector, and *e* is the error vector.

The LS-SVM regression function can be written as:


$$\begin{array}{c} f(x)=\sum {\mathrm{\backslash nolimits}}_{i=\text{1}}^{n}\alpha_{i}K\left({x,\;x}_{i}\right)+b \end{array}$$ (7)

where *x* denotes the independent variable, α_i_ are the weight coefficients associated with the support vectors, and K(*x,x*_i_) represents the kernel function. The bias term *b* acts as an intercept, shifting the regression function vertically in the feature space to improve flexibility and reduce bias. Both α_i_ and *b* are optimized simultaneously during the training process to minimize prediction error.

Kernel functions map input data into a higher-dimensional space, allowing the model to capture complex relationships. Four kernel functions were tested in this study to identify the best LS-SVM model: radial basis function (RBF), linear, polynomial degree 2 (Poly2), and polynomial degree 3 (Poly3).

Before modeling, the data were preprocessed using z-score normalization and split into training and testing sets. The LS-SVM models were trained on the training data, with parameters tuned to maximize predictive performance. All analyses were conducted using MATLAB software.

In this study, three independent variables were investigated: MC, EBRT, and inlet H₂S concentration. Each variable was tested at three levels to cover a wide range of operating conditions: MC at 30%, 70%, and 90% (wet basis); EBRT at 20, 40, and 60 s; and inlet H₂S concentration at 97.5 and 180 ppmv. A full factorial design was applied, resulting in 27 unique combinations (3 × 3 × 3). Each combination was performed in triplicate, yielding a total of 81 experimental runs. This design allowed the assessment of both main effects and interactions while ensuring sufficient data variability for reliable model development. For modeling, the complete dataset was randomly divided into training (80%) and validation (20%) subsets. This approach ensured that both the SVM and MLR models were trained on representative conditions while reserving independent data for unbiased validation. The resulting dataset provides a statistically balanced foundation for evaluating predictive performance and identifying optimal conditions for H₂S removal.

### 2.6. Optimizing H_2_S removal in a biofilter with genetic algorithm (GA)

The genetic algorithm (GA) is an optimization method inspired by natural selection and genetic inheritance. It starts by generating a random population of candidate solutions, where each solution represents a unique combination of bed moisture content (MC) and empty bed residence time (EBRT). The performance of each candidate is evaluated using a fitness function, which in this study was based on predictions from either the multiple linear regression (MLR) model or the support vector machine (SVM) model.

**Fig 3:**
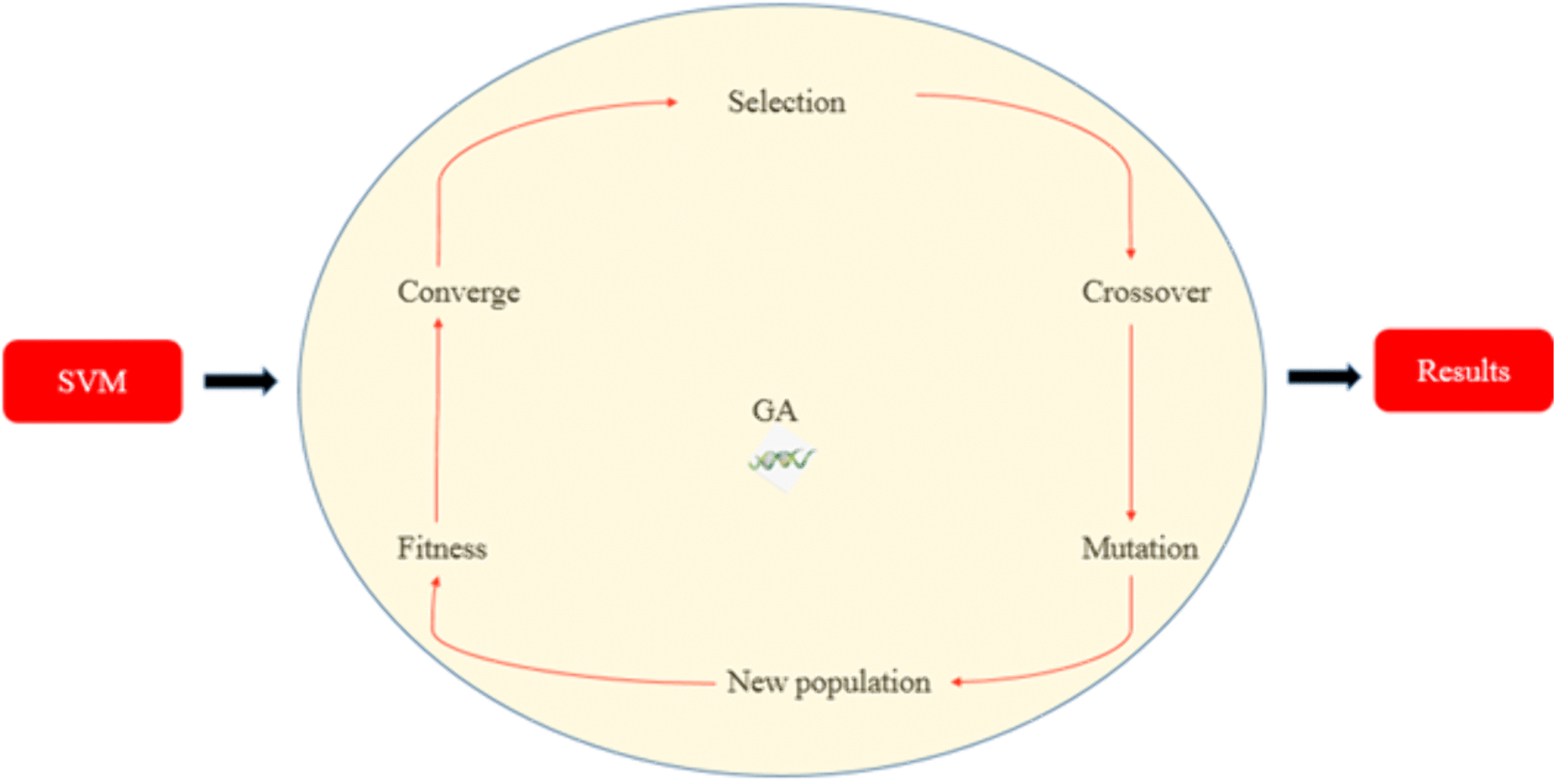
Flowchart of the genetic algorithm (GA) approach used to optimize bed moisture content (MC) and empty bed residence time (EBRT) for efficient hydrogen sulfide (H_2_S) removal in a biofilter containing biochar.

The GA procedure involves several steps, as shown in Fig 3. First, fitness scores are calculated for all candidates. The selection operator then chooses the best-performing individuals to form the next generation. Diversity is maintained using two genetic operators: crossover, which combines pairs of candidates to create new solutions, and mutation, which introduces random variations. This cycle of selection, crossover, and mutation continues for a predefined number of generations. In this study, the GA ran for 200 generations. The candidate with the highest fitness score at the end of the process was selected as the optimal combination of MC and EBRT for maximizing H₂S removal efficiency.

Table 2 summarizes the GA parameters, including population size, number of generations, crossover and mutation rates, selection and migration methods, and the ranges of MC and EBRT. All GA computations were performed in MATLAB.

**Table 2:** Summary of the genetic algorithm (GA) properties.

Value/probability	Type	Parameter
100	–	Population size
200	–	Number of generations
–	Scattered	Crossover
0.01	Uniform	Mutation
–	Uniform	Selection
0.20	Forward	Migration

### 2.7. Performance evaluation criteria for the MLR and SVM models

The performance of the multiple linear regression (MLR) and support vector machine (SVM) models in predicting H₂S removal efficiency (RE) in the biochar-packed biofilter was assessed using two metrics: the coefficient of determination (R²) and root mean square error (RMSE). R² quantifies the proportion of variance in the observed data explained by the model predictions and is calculated as:


$$R^{\text{2}}=\frac{(\sum_{i=\text{1}}^{n}{\left(x_{o.i}-{\overset{―}{x}}_{o})(x_{p.i}-{\overset{―}{x}}_{p})\right)}^{\text{2}}}{\sum_{i=\text{1}}^{n}{\left(x_{o.i}-{\overset{―}{x}}_{o}\right)}^{\text{2}}\times \sum_{i=\text{1}}^{n}{\left(x_{p.i}-{\overset{―}{x}}_{p}\right)}^{\text{2}}}$$ (8)

RMSE measures the average difference between observed and predicted values and is defined as:


$$RMSE=\sqrt{\frac{\sum_{i=\text{1}}^{n}{\left(x_{o.i}-x_{p,i}\right)}^{\text{2}}}{n}}$$ (9)

where $x_{o}$ and $x_{p}$ are the observed and predicted values, respectively, and ${\overset{―}{x}}_{o}$ and ${\overset{―}{x}}_{p}$ are their corresponding means.

In this study, the model with the highest R² and lowest RMSE was considered the best. MLR and SVM modeling were performed using MATLAB R2019a, while descriptive statistics and data analysis were conducted using Minitab 21.3.1.

## 3. Results and discussion

Hydrogen sulfide levels in biogas vary widely, from trace amounts below 50 ppmv to more than 10,000 ppmv in extreme cases. In the present study, a concentration range of 97–180 ppmv was examined because it corresponds to the levels naturally produced during anaerobic digestion under the operating conditions of the experimental system. This range was therefore considered the most realistic for assessing biofilter performance. Future research should investigate both lower (<50 ppmv) and higher (>1000 ppmv) concentrations to better capture the variability encountered in practical biogas applications.

### 3.1. Factors affecting the efficiency of H_2_S removal in biofilters containing biochar

The inlet concentration of H₂S ranged from 180 to 97 ppmv, while the outlet concentration ranged from 15 to 9 ppmv. This corresponds to removal efficiencies between 85% and 95%. Throughout the experiment, no noticeable changes were observed in the CH₄ concentration. To evaluate the effects of operating parameters, an analysis of variance was conducted for three factors: bed moisture content (MC), empty bed residence time (EBRT), and inlet H₂S concentration. As presented in Table 3, all three factors had significant main effects (p < 0.01), and the interaction between EBRT and MC was also significant. These results indicate that the combined influence of moisture content and residence time plays a critical role in determining the overall efficiency of H₂S removal in biochar-packed biofilters.

**Table 3:** ANOVA results for bed moisture content, empty bed residence time, and H_2_S input concentration.

Source of Variation	DF	SS	MS	F-Value	p-value
MC	2	1011.68	505.84	852.43	0.00
EBRT	2	2843.61	1421.80	2396.01	0.00
H_2_S_IN	1	186.01	186.01	313.46	0.00
MC×EBRT	4	1307.27	326.82	550.75	0.00
Error	44	26.11	0.59		
Total	53	5374.67			

Note: DF represents degrees of freedom, SS represents sum of squares, and MS represents mean squares.

**Fig 4:**
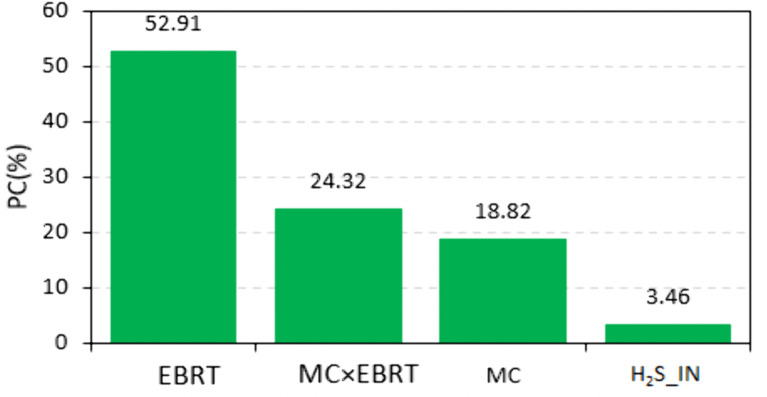
Percent contribution (PC) of key factors to H_2_S removal efficiency in biofilters containing biochar.

The contribution of each factor to H₂S removal efficiency in the biochar-packed biofilter is presented in Fig 4. The percent contribution (PC) was determined by dividing the sum of squares of each factor by the total sum of squares. Results showed that EBRT had the largest contribution, followed by the interaction of EBRT and MC, then MC, and finally the inlet H₂S concentration. These findings indicate that the combined effect of MC and EBRT plays a crucial role in enhancing H₂S removal efficiency. Similar trends have been reported in previous studies [34,44]. Overall, the results emphasize the importance of MC and EBRT as key parameters in the design and optimization of biochar-based biofilters for effective H₂S removal.

**Fig 5:**
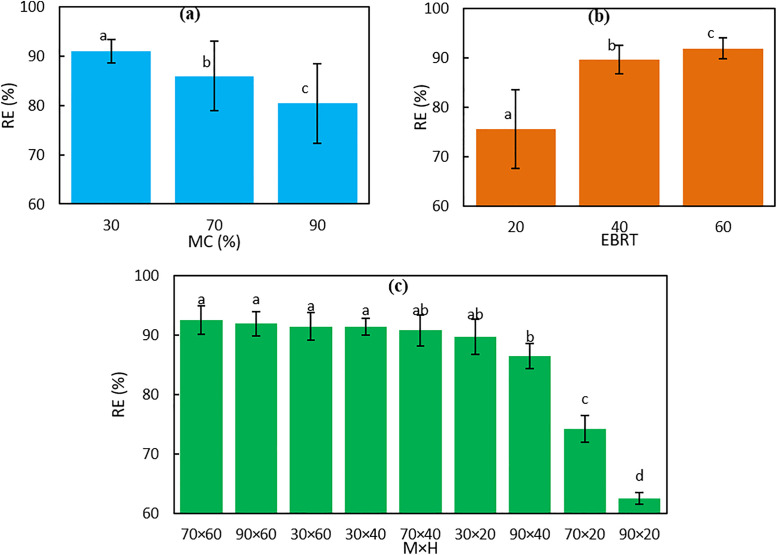
Statistical comparison of different levels of factors affecting H_2_S removal efficiency (RE) by least significant difference method (LSD) at a 5% significance level.

Fig 5 shows the results of the least significant difference (LSD) test at a 5% significance level, which was used to evaluate the effects of bed moisture content (MC) and empty bed residence time (EBRT) on H₂S removal efficiency (RE) in the biofilter. Fig 5a demonstrates that RE decreases significantly as MC increases. The mean RE was highest at 30% MC with 90.97%, followed by 70% MC at 85.93%, and lowest at 90% MC with 80.37%. Each MC level belonged to a distinct statistical group, indicating that higher moisture content negatively affects biofiltration efficiency. Low MC facilitates better interaction between the gas and biochar, requires less water, and is easier to maintain, consistent with previous studies on the treatment of low-concentration polluted gases [45]. Fig 5b illustrates the influence of EBRT on RE. Increasing EBRT from 20 to 60 seconds led to a significant rise in the mean RE from 75.58% to 91.96%, with each EBRT level forming a separate statistical group. The higher RE observed at longer EBRTs is likely due to prolonged contact time, which enhances the adsorption and biodegradation of H₂S, in agreement with earlier reports [46]. The interaction between MC and EBRT is shown in Fig 5c. High RE values above 91% were observed when low to moderate MC levels were combined with long EBRTs (30 × 60, 30 × 40, 70 × 60, 90 × 60), all grouped statistically as the highest-performing combinations. Conversely, high MC (90%) combined with short EBRT (20 s) resulted in the lowest RE of 62.5%, forming a separate statistical group. These findings indicate that the adverse effect of high MC can be partially offset by increasing EBRT, while short EBRT under high MC conditions severely reduces H₂S removal.

The LSD analysis confirms that both MC and EBRT significantly influence H₂S removal efficiency, and their interaction plays a critical role in optimizing biofilter performance. The results highlight the effectiveness of biochar as a packing material, providing high removal efficiency under optimal operating conditions. These observations are consistent with previous studies reporting high H₂S removal using biochar and other substrates such as compost and peat [20,44,47,48]. The high porosity, availability, and cost-effectiveness of biochar make it particularly suitable for biofilters treating low-concentration H₂S.

### 3.2. Modeling H_2_S removal efficiency using multiple linear regression (MLR)

Table 4 shows the analysis of variance (ANOVA) results for the multiple linear regression (MLR) model used to predict H₂S removal efficiency (RE). Stepwise regression was applied, and non-significant terms were excluded from the final model. The results indicate that the regression model was highly significant at the 1% level, confirming that RE was strongly influenced by the main operational factors of the biofiltration process. The model achieved a coefficient of determination (R²) of 96.56%, with adjusted and predicted R² values of 96.13% and 95.49%, respectively. These results demonstrate that the selected variables provided consistent and accurate predictions of H₂S RE.

**Table 4:** Analysis of variance (ANOVA) results for multiple linear regression (MLR) model.

Source of Variation	DF	Seq SS	Adj MS	F-Value	p-value
Regression	6	5190.01	865	220.15	0.00
MC	1	963.85	51.12	13.01	0.00
EBRT	1	2416.38	203.82	51.87	0.00
H_2_S_IN	1	186.01	186.01	47.34	0.00
MC × MC	1	47.82	47.82	12.17	0.00
EBRT×EBRT	1	427.23	427.23	108.73	0.00
MC×EBRT	1	1148.71	1148.71	292.36	0.00
Error	47	184.67	3.93		
Lack-of-Fit	11	174.21	15.84	54.53	0.00
Pure Error	36	10.46	0.29		
Total	53	5374.67			

Note: Seq SS refers to Sequential Sum of Squares; Adj MS refers to Adjusted Mean Squares.

Although the MLR model explained more than 96% of the variability, the ANOVA results also revealed a significant lack-of-fit (p < 0.01). This indicates that the model did not fully capture the true relationships between operational variables and RE. In regression analysis, a significant lack-of-fit suggests that residual variation is not only due to random error but also to model misspecification. Consequently, a high R² alone does not ensure reliable prediction, particularly when nonlinear interactions exist. To address this limitation, a least-squares support vector machine (LS-SVM) was applied. LS-SVM can effectively model nonlinear and complex interactions without strict linear assumptions and performs well with relatively small datasets. Compared with conventional regression, it provides stronger generalization and greater flexibility, making it a more suitable tool for predicting H₂S removal efficiency in biofiltration systems.

**Fig 6:**
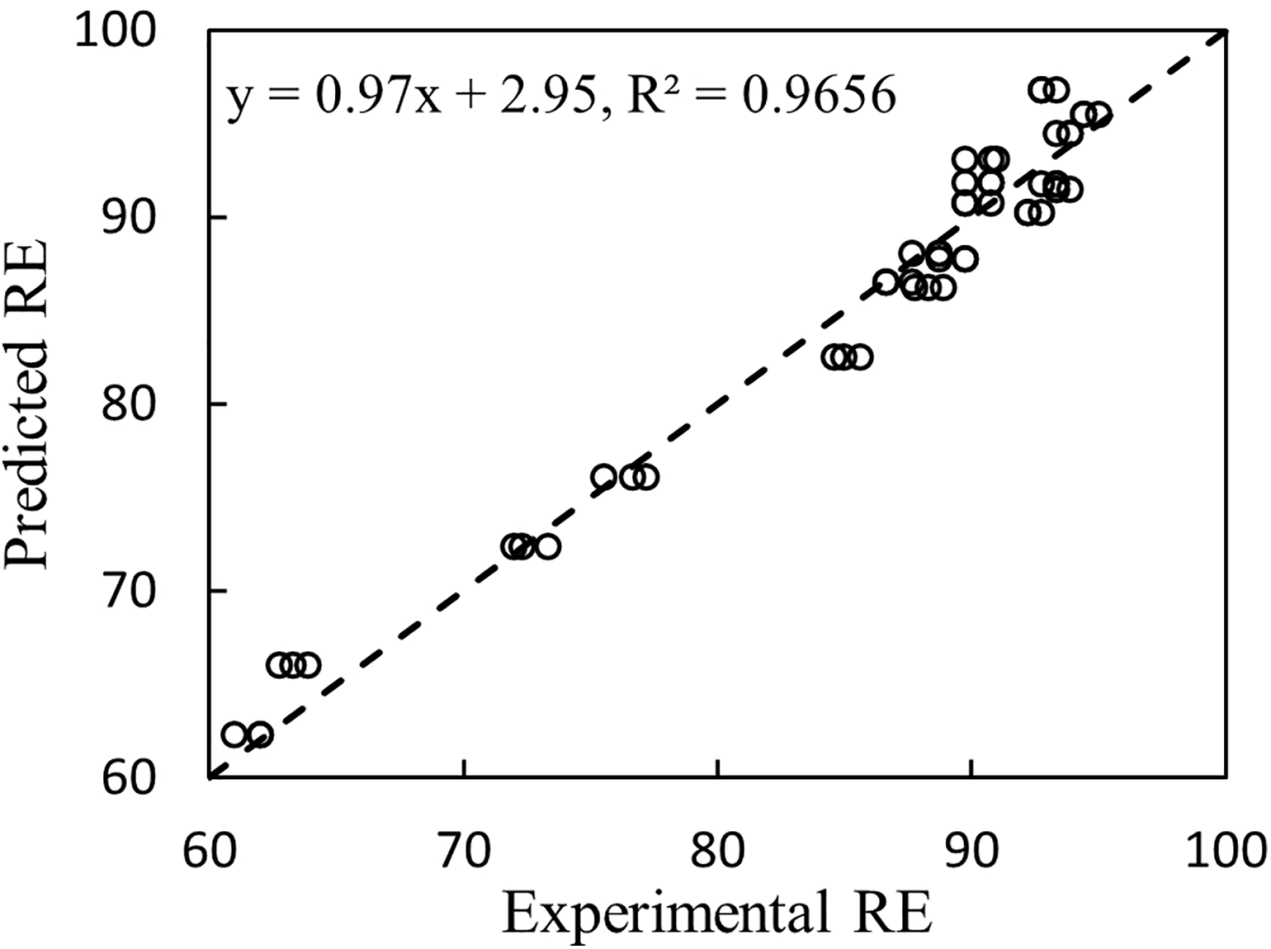
Comparison of MLR model predictions with experimental data.

Fig 6 illustrates the comparison between the predicted data obtained from the MLR model and the experimental data. The high value of R^2^, close proximity of the line’s slope to 1, and the y-intercept’s proximity to zero suggest that the two sets of data are in good agreement with each other.

**Fig 7:**
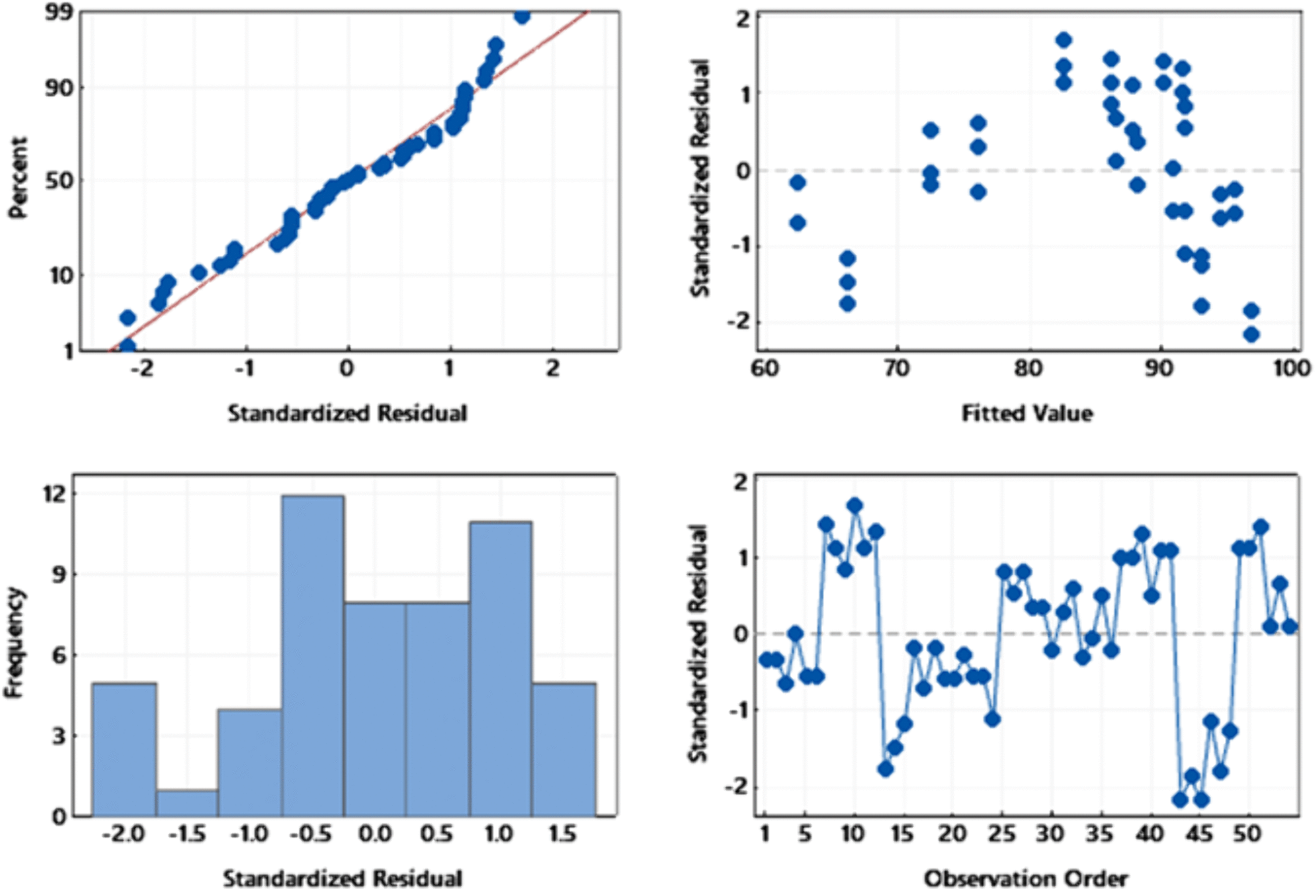
Multiple linear regression (MLR) model diagnostics for H_2_S removal efficiency.

Fig 7 presents the results of the diagnostic tests carried out to assess the assumptions of the MLR model, including normality of the model error distribution, independence of the error variance, and absence of error autocorrelation. Fig 7(a) and 7(c) demonstrate that the model error distribution follows the normal distribution, and this finding is further supported by the Kolmogorov-Smirnov test with a p-value of 0.42. The absence of a clear pattern in the change pattern observed in Fig 7(b) confirms the assumption of independence of the MLR model error. Fig 7(d) indicates that the MLR model errors do not exhibit autocorrelation, as the distribution of experimental errors does not follow any discernible trend.

### 3.3. H_2_S removal efficiency modeling using support vector machine (SVM)

In this study, the least squares support vector machine (LS-SVM) method was utilized as an alternative approach to model the H_2_S elimination process. Identifying the type of kernel function is a critical parameter in the design of SVM models. Table 5 presents a comparison of the performance of four kernel functions, including Radial Basis Function (RBF), polynomial degree 1 (poly1), polynomial degree 2 (poly2), and polynomial degree 3 (poly3), in modeling the RE of H_2_S in the biofilter. According to the results, the polynomial degree 3 kernel function demonstrated the best performance in predicting H_2_S RE. The R^2^ and RMSE values for the training, testing, and total datasets were 0.99 and 0.6, respectively. For this study, 80% of the total data was randomly selected to train the SVM model, while the remaining 20% was utilized to test the model.

**Table 5:** Evaluation of kernel function varieties in support vector machine (SVM) for modeling H_2_S removal efficiencies.

	Train		Test		Total	
Kernel function	RMSE	R^2^	RMSE	R^2^	RMSE	R^2^
Poly1	5.78	0.6932	6.07	0.5741	5.84	0.6621
Poly2	1.18	0.9746	2.41	0.9435	1.95	0.9635
Poly3	0.6	0.9934	0.59	0.9942	0.6	0.9933
RBF	0.79	0.9954	1.11	0.9963	0.87	0.9964

**Fig 8:**
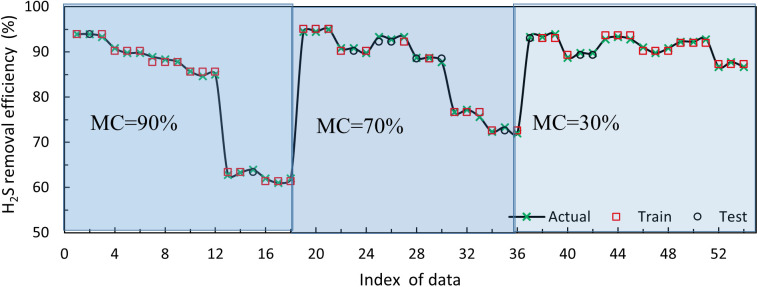
Comparison of experimental data with SVM model predictions for training, testing, and total datasets.

Fig 8 displays a comparison between the experimental data and the data obtained from the SVM model for predicting H_2_S removal efficiency in the biofilter for the training, testing, and total datasets. The model exhibited acceptable accuracy in predicting the RE in all three sets, as shown in the figure. The high R^2^ values observed in all stages of training, testing, and total indicate that the proposed models were well-trained and precise in predicting H_2_S removal efficiency.

Train: y = 0.99x + 0.45, R^2^ = 0.9954, Test: y = 1.00x-0.92, R^2^ = 0.9963, Total: y = 0.99x + 0.28, R^2^ = 0.9964

To evaluate the generalizability of the SVM model, training and testing stages were performed using varying sizes of training sets ranging from 50% to 90% of the total dataset in this study. Table 6 displays the results of this study in terms of changes in the two criteria, RMSE and R^2^, for the training, testing, and total stages. The results indicate that as the training set size decreases and the test set size increases, the RMSE value increases, but the R^2^ value remains nearly constant, as shown in Table 5. The SVM model can make acceptable predictions for up to 60% of the training set, indicating that its generalizability is confirmed. Furthermore, since SVM does not have overfitting, it can be utilized as a reliable model for predicting H_2_S removal efficiency based on biofiltration process parameters.

**Table 6:** Evaluation of support vector machine (SVM) model generalizability for different data ratios in three phases of training, testing, and total.

	Train		Test		Total	
Train Size (%)	RMSE	R^2^	RMSE	R^2^	RMSE	R^2^
90	0.6	0.9954	0.46	0.9963	0.6	0.9964
80	0.6	0.9934	0.59	0.9952	0.6	0.9963
70	0.6	0.9945	0.75	0.9943	0.65	0.9954
60	0.6	0.9953	0.9	0.9947	0.73	0.9943
50	0.53	0.9954	1.64	0.9754	1.22	0.9832

### 3.4. Optimization of H_2_S removal efficiency using genetic algorithm

To optimize H₂S removal efficiency, three parameters were considered: bed moisture content (MC), empty bed residence time (EBRT), and H₂S input concentration. For this purpose, either the Multiple Linear Regression (MLR) model or the Support Vector Machine (SVM) model could be used as the fitness function in the genetic algorithm (GA). A comparison of the models showed that the SVM model provided more reliable predictions than the MLR model. The MLR model exhibited a significant lack of fit, whereas the SVM model achieved a higher coefficient of determination (0.9964) compared to the MLR model (0.9656) for both experimental and predicted datasets (Figs 6 and 8). In addition, while the MLR model was trained using the entire dataset, the SVM model was developed with 80% of the data, further supporting its robustness. Therefore, the SVM model was selected as the basis for GA optimization.

During optimization, the H₂S input concentration was maintained within the range of 97–180 ppmv, as this parameter is controlled by the output of the biogas digester. The GA was then applied to identify the optimal values of bed MC and EBRT that maximize H₂S removal efficiency. The optimized values for different H₂S input concentrations are summarized in Table 7. The results show that the optimal levels of bed MC and EBRT depend on the H₂S input concentration, and the removal efficiency ranged between 90% and 95%. Experimental validation confirmed that the treatment with 70% bed moisture content and 60 seconds EBRT achieved an average removal efficiency of 92.53 ± 2.34%, with a maximum observed value of 95% at an input concentration of 180 ppmv. These results highlight the need for an operational control strategy in which the biofilter settings are adjusted according to the H₂S input concentration. Fixed values of bed MC and EBRT are not sufficient, since the optimal conditions vary with the inlet concentration. On average, the optimal values of bed MC and EBRT were 54% and 41 seconds, respectively. Nevertheless, the efficiency of a biofilter based solely on these two parameters is unlikely to exceed 90–95%. Further improvement may require optimization of additional operating factors such as temperature, pH, and the use of substrate additives.

**Table 7:** Optimization of bed MC and EBRT for maximizing H_2_S removal efficiency at various input concentrations using genetic algorithm.

Optimization	MC	EBRT	H_2_S_IN	RE (%)
Genetic algorithm	77.39	52.56	104	90.64
	77.09	51.28	110	90.66
	30.00	27.28	134	91.64
	30.00	26.96	146	92.08
	30.00	27.79	158	92.52
	71.19	55.54	176	94.29
	70.72	58.36	180	95.05
Experimental	70	60	180	92.53 ± 2.34

**Fig 9:**
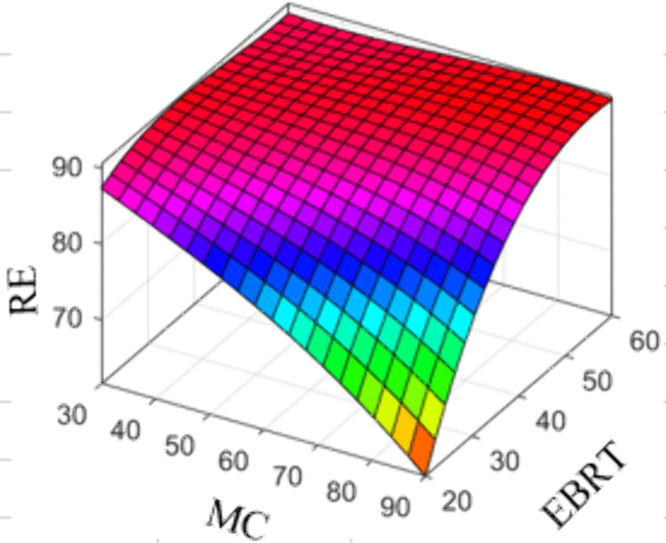
Response surface plot of H_2_S removal efficiency as a function of bed MC and EBRT for H_2_S input concentration of 180 ppmv.

Fig 9 displays the response surface plot of H_2_S RE as a function of changes in bed MC and EBRT for an H_2_S concentration of 180 ppmv, generated using the SVM model. This figure was used to investigate and comprehend the changes in H_2_S RE in the biofilter. The response surface plot in Fig 9 illustrates a clear interaction between bed MC and EBRT on H_2_S RE. At different levels of bed MC, increasing the RE for an EBRT of more than approximately 40 seconds can result in a range of 90% to 95%. Depending on the EBRT, a 55% reduction in bed MC can lead to higher removal efficiency at a high EBRT.

## 4. Conclusion

In summary, this study examined the effects of bed moisture content (MC), empty bed residence time (EBRT), and inlet H₂S concentration on biofilter removal efficiency (RE) using variance analysis and predictive modeling with multiple linear regression (MLR) and support vector machines (SVM) combined with genetic algorithm (GA) optimization. Laboratory experiments showed that the biofilter achieved high removal efficiencies, with a maximum of 95% under optimal MC and EBRT conditions. Variance analysis confirmed that all main effects and the interaction between MC and EBRT were significant at the 99% level, with EBRT contributing the most to H₂S removal. Both MLR and SVM provided accurate predictions of removal efficiency, although SVM showed superior performance. The optimization results further indicated that the best values of MC and EBRT varied with inlet H₂S concentration. For future studies, incorporating additional variables such as temperature and microbial activity into biofilter models may further enhance predictive accuracy and removal performance.

This study focused on H₂S removal within a concentration range of 97–180 ppmv. Although the developed models reliably predicted removal efficiency in biochar-packed biofilters under these conditions, their applicability to other packing materials or wider concentration ranges remains unverified. Future studies should expand this framework to include lower (<50 ppmv) and higher (>1000 ppmv) concentrations and validate the models through pilot-scale experiments or alternative biofilter configurations to fully capture the variability of biogas systems.

### Nomenclature

**Table:** 

ANOVA	Analysis Of Variance, (-)
C_H2S_,in	Concentration of H_2_S input to the biofilter, (ppmv)
EBRT	Empty Bed Residence Time, (s^-1^)
GA	Genetic Algorithm, (-)
LOF	Lock of Fit, (-)
LS	Least Squared, (-)
LSD	Least Significant Difference, (-)
MC	Moisture Content, (%)
ML	Machine Learning, (-)
MLR	Multiple Linear Regression, (-)
Poly 1	Polynomial Degree 1, (-)
Poly 2	Polynomial Degree 2, (-)
Poly 3	Polynomial Degree 3, (-)
PVC	Polyvinyl Chloride, (-)
R^2^	R-squared, (-)
RBF	Radial Bias Function, (-)
RE	Removal Efficiency, (%)
RMSE	Root Mean Square Error, (-)
SVM	Support Vector Machine, (-)

## Supporting information

S1 Fig Graphical abstracts. (TIF)
